
JOURNAL INFORMATION
==============================
NLM Title Abbreviation: ScientificWorldJournal
Journal ID: ScientificWorldJournal
NLM Journal ID: 101131163
Journal ID: TSWJ

The Scientific World Journal

ISSN: 2356-6140
EISSN: 1537-744X
Publisher: Wiley

ARTICLE INFORMATION
==============================
PMCID: PMC5974742
DOI: 10.1100/tsw.2003.20
Article ID: 876067
Article version: 1
Subjects: Book Review

Book Review "Childhoods in Context"

Merrick Joav *
National Institute of Child Health and Human Development, Office of the Medical Director, Division for Mental Retardation, Ministry of Social Affairs, Box 1260, IL-91012 Jerusalem, Israel
*Joav Merrick: jmerrick@internet-zahav.net
Publication date: 2003
Electronic publication date: 2003 Apr 14
Volume: 3
First page: 239
Last page: 239
Copyright: Copyright © 2003 Joav Merrick.
Copyright year: 2003
License: This is an open access article distributed under the Creative Commons Attribution License, which permits unrestricted use, distribution, and reproduction in any medium, provided the original work is properly cited.
License URL: https://creativecommons.org/licenses/by/3.0/

==============================
Book Review
*TheScientificWorldJOURNAL* (2003) 3, 239
ISSN 1537-744X; DOI 10.1100/tsw.2003.20

### Book Review — Childhoods in Context

Joav Merrick, M.D.
*National Institute of Child Health and Human Development, Office of the Medical Director, Division for Mental Retardation, Ministry of Social Affairs, Box 1260, IL-91012 Jerusalem, Israel*

**Email:** jmerrick@internet-zahav.net

*Received March 28, 2003; Accepted March 28, 2003; Published April 14, 2003*

---

**Maybin, J. and Woodhead, M., Eds. (2003) *Childhoods in Context*. John Wiley & Sons and Open University, Chichester. 326 pages. Price: 45.00 US$ (softcover).**

**DOMAINS:** child health and human development

---

This book is the second in a series of four in a new project between John Wiley and the Open University and is based on the course on "Childhood" at that university. The editors and the seven contributors are primarily affiliated with the Centre for Childhood, Development and Learning at the Open University and with this second boo in the series the appreciation of reading gets even better.

This colorful book takes you from children growing up in a fishing community in 19th century Sweden to childhood in Bolivia and Zimbabwe; child work in Bangladesh; and children at school in England, Japan, and India. The transition to adulthood — especially the transition of children or adolescents with a disability — is also touched upon. You will find useful comments, further reading suggestions, and many questions asked throughout the text.

The book has seven extensive chapters on the socializing of children, family and kinship, child life from 1800–2000, children and school, children and work, early childhood education, and moving out of childhood.

The topic and history of adolescence are also discussed, relying on the influence of the American psychologist G. Stanley Hall, who published his book *Adolescence* in 1904. In this book, adolescence was described as the age range of 14–25 years. In the same time period, youth movements such as the Boy's Brigade (started in Glasgow in the 1880s) became very popular. These movements (later Boy and Girl Scouts, etc.) have been so successful that many children have joined and it is believed that, in many ways, the introduction of adolescents to these movements has helped to prolong childhood.

Each chapter in this book was written in a clear, straightforward language; the lay-out is wonderful; and this book can be warmly recommended to everyone who works with children.

---

**This article should be referenced as follows**:

Merrick, J. (2003) Book review — Childhoods in Context. *TheScientificWorldJOURNAL* **3**, 239

---

©2003 with author.
